# Simulation translation differences between craft groups

**DOI:** 10.1186/s41077-022-00218-z

**Published:** 2022-07-27

**Authors:** Jye Gard, Chi Duong, Kirsty Murtagh, Jessica Gill, Katherine Lambe, Ian Summers

**Affiliations:** 1 Monash University, Melbourne, Australia; 2 Monash Centre for Scholarship in Health Education (MCSHE), Faculty of Medicine, Nursing and Health Sciences, Monash University, Melbourne, Australia; 3 Monash Simulation, Moorabbin Hospital, Melbourne, Australia

**Keywords:** Delivery of healthcare, Medical simulation, Interprofessional learning, Clinical Translation Science

## Abstract

**Background:**

Many simulation-based clinical education events (SBCEE) aim to prepare healthcare professionals with the knowledge, skills, and features of professionalism needed to deliver quality patient care. However, how these SBCEE learnings are translated into broader workplace practices by learners from different craft groups has not been described.

**Objectives:**

To understand how learners from different craft groups (doctors and nurses) anticipate simulation-based learnings will translate to their workplaces and the process by which translation occurs.

**Design:**

Qualitative descriptive study design using pre- and post-SBCEE questionnaires.

**Settings:**

A large tertiary Australian hospital-based simulation centre that facilitates SBCEE for multi-professional graduate and undergraduate clinicians from 16 hospitals.

**Methods:**

Participants who attended SBCEEs between May and October 2021 completed questionnaires at two touchpoints, on the day of attending a SBCEE and 6 weeks after. Based on a phenomenological approach, the study examined clinicians’ experiences in relation to simulation education, intended simulation learning use in the workplace, and perceived success in subsequently using these learnings to improve clinical outcomes. Qualitative inductive thematic data analysis was used to develop narratives for different learner cohorts.

**Results:**

Three overarching themes were identified regarding simulation participants’ perceptions of the success of translating simulation learnings into the workplace. These were: scenario-workplace mirroring, self-assessment, and successful confidence. Doctor participants found it difficult to map SBCEE learnings to their workplace environments if they did not mirror those used in simulation. Nurses sought peer evaluation to analyse the effectiveness of their workplace translations, whereas doctors relied on self-assessment. Learners from both craft groups highly prized ‘confidence-building’ as a key indicator of improved workplace performance achieved through SBCEE learning.

**Conclusion:**

A diverse range of factors influences healthcare workers’ experiences in translating simulation learnings to their workplace. To equip simulation learners to translate learnings from a SBCEE into their clinical practices, we suggest the following areas of focus: co-development of translation plans with learners during the delivery of an SBCEE including the indicators of success, above table discussions on the generalisability of learnings to different environments and contexts, smart investment in simulation outputs, and cautious championing of confidence-building.

## Background

The goal of many simulation-based clinical education events (SBCEE) is to prepare healthcare professionals with the knowledge, skills and features of professionalism needed to deliver quality patient care [1]. The simulation educator does this by engaging learners in a controlled experiential event designed to mimic a clinical encounter, then facilitating a learning conversation to promote reflection on mental models and behaviours [2]. Translational science has shown that SBCEE programs may improve knowledge retention, reproduce specific procedural skills, and ignite behavioural change in certain clinical settings [3–9]. However, there is a relative paucity of data on whether SBCEE learnings may be translated into broader workplace practices and their impact on clinical outcomes, particularly in Australian workplace settings [5].

Providing evidence for the clinical impact of an SBCEE is difficult [6, 10, 11]. Classically, the close mapping of an SBCEE to a ‘true’ clinical event together with quality simulation methodology was assumed to result in improved learner workplace practices [5]. However these assumptions are usually flawed, as the outputs expected from an SBCEE are often broad, complex or ill-defined [6]. Commonly an SBCEE is developed as an intervention to change a specific health service performance indicator (e.g. reduce the total number of adverse events associated with a specific surgery) [6]. However unlike other types of interventions, SBCEEs are often created without also mapping the additional success indicators, workplace barriers and enablers that need to be monitored or addressed to ensure implementation translates into improved clinical output [6]. Translational simulation is a concept that aims to overcome this gap by helping simulation facilitators develop and assess explicit outputs for SBCEEs [5]. Translational simulation harnesses the considerate crafting of an optimal learning environment, close stakeholder enmeshment, and a wider consideration of health system levers, to develop and deliver a staged SBCEE to achieve detailed outcomes [5, 12]. Another cornerstone of translational simulation is the act of analysing the impact of a SBCEE and distributing these findings to key hospital education stakeholders [5]. Thus, the act of a health service studying its translation simulation activities itself enhances the chances of workplace adaptation [5].

Whilst this systems-based approach provides a framework for SBCEE interventions to affect change in healthcare institutions, it largely omits the individual learner narratives surrounding the process of implementation and guidance on how they themselves might act as agents for change. Furthermore, most SBCEEs are delivered to groups of diverse learners; healthcare teams with staff of different craft groups, levels of seniority and experience. Thus, further research is urgently needed to help identify the specific needs different cohorts of learners require to translate their SBCEE learnings into improved clinical outcomes.

Hence this study aimed to understand the “lived-experience” of clinicians who attend a simulation-based education event and how these learnings are translated to the clinical workplace.

## Methods

### Study design

The study used an explorative qualitative design to explore the cohort narrative [13]. The “lived-experience” of SBCEE learners were drawn from thematic analysis of the learning reflections of participants via thematic analysis of questionnaires across two-time points (Table 1). The study used the lens of the phenomenological approach [14], to understand *what* learners experienced and *how*, gaining a deeper understanding of the nature or meaning of the SBCEE experience phenomena.

**Table 1:** Questionnaire

Q1: First questionnaire (Immediately following SBCEE)	Q2: Second questionnaire (8 weeks post-SBCEE)
Please list three things you learnt from this course that you will use in your clinical environment	What was the date you completed your course with Monash Simulation?
How often do you expect to use these skills/learnings?	Since the course, how often have you used the skills or learnings?
Over the next year, how will you be able to tell if today’s learning has changed your clinical practice?	Is this more or less than expected? (Likert scale)
	If you were going to further upskill in this area, what is the next step you could take?
	Please tell us about either: A time you have used the learnings from your Monash Simulation course or Why have you not had the opportunity to use your Monash Simulation course learnings at work?

### Study settings

The study recruited from a single Victorian hospital-based simulation centre that provides 16 different SBCEE types to a variety of learners from ward, emergency and theatre-based roles. These events were attended by both clinicians and health professions from a variety of Victorian health networks. During COVID-19 restrictions, on average 40 participants attended the simulation centre each week. All learners who attended an education event at the simulation centre between May to October 2021 were eligible for participation.

### Participant characteristics

SBCEE comprised of learners from different craft groups and job roles who work across various health sites, including medical students, nursing students, trainee doctors from multiple subspecialties and levels of seniority, graduate nurses from multiple subspecialties and level of seniority, consultant doctors and nurse unit managers.

### Recruitment

Participants were identified via an opt-in process. Depending on the number of volunteers, the researchers purposively selected a mix of participants from different worksites to capture differences in education impacts experienced in different roles. Once ethics approval was attained, participants were recruited on completion of all Monash Simulation Centre education events.

### Simulation-Based Clinical Education Events (SBCEE)

The simulation centre facilitated 16 different types of SBCEE: each whole day events with different aims and content of workshops and simulation scenarios. Twelve event types were opt-in and occurred outside rostered teaching time. The details of each differed to align with learning needs, the scope of practice and local protocols applicable to the direct clinical management of patients in different learners’ settings. However, there was also significant overlap of content between sessions, as each type of event focussed on discussing how human factors influence our ability to work as a team during high stress events. Participants then had the opportunity to practice these principles with Inter-professional simulated scenarios, each one followed by a debrief and discussion to explore the team performance.

### Data collection

The study was conducted via questionnaires at two-time points: immediately after completing the simulation session (Q1) and again 8 weeks later (Q2) to ensure time to trial the learnings in the workplace after participation in a SBCEE. The questionnaires were distributed and collected by the administrative staff organising the SBCEE. These were completed on-site. The second survey was accessed via a QR code sent via letter together with the participant's first questionnaire responses to prompt reflection on intended workplace translation (Table 1). All questionnaire responses were converted into electronic documents and used for analysis.

### Data analysis and reflexivity

Questionnaires were coded independently by two investigators (JG and IS) following an iterative process with comparison. Coding was conducted using NVivo, Release 1.4 (QSR International). These were reviewed by JG and IS and further discussed as a team to compare, contrast and negotiate team-based interpretations of the themes and sub-themes generated. The discussions included a notion of reflexivity where the researchers examined their positioning within the research. Narrative analysis (was then performed on the coded data through the lens of Bruner’s functional approach [1].

## Results

### Professional characteristics

A total of 226 participants attended SBCEEs during the study period. Two hundred six participants completed the first questionnaire (participation rate 91%). Sixty-seven completed both questionnaires (participation rate 30%) consisting of 37 doctors, 26 nurses and 4 students. Their years of experience ranged from 1 to 51 years, with a median of 12 years. Participants held different job roles and worked across various health sites and subspecialties.

### Themes

The qualitative data from questionnaires (Table 1) was analysed using inductive content analysis. Three central themes regarding the translation of simulation learnings to the workplace were identified: scenario-workplace mirroring, self-assessment and successful confidence.

### Scenario-workplace mirroring

Whilst the content of some of the SBCEE differed, the technical skill teachings, settings, and contexts were tailored to learner groups, other teaching topics were common to all sessions, including crisis resource management, optimising interprofessional teamwork, communication skills and management of cognitive load. Immediately following the SBCEE, many learners identified these universal teachings as key, and their successful use in day-to-day practice across all settings would result in measurable change.

*“I will know I have improved if I:*

1. *Use the communication strategies within a team.*
2. *Am recognising cognitive overload & [using] de-stressing strategies” (Nurse, Participant 36, Q1)*

However, many doctor participants later reported difficulty in applying these SBCEE learnings to work unless they had encountered a clinical situation that mirrored the specific setting and context of the simulation sessions.

*“Had a lot of cover shifts so have not been dealing with a lot of the intraoperative issues that we tackled in the simulation session...” (Doctor, Participant 24, Q2)*

This contrasted with most of the nurse participants who more readily identified opportunities to use simulation learnings, regardless of their workplace setting or role.

*“Beneficial in real scenario-witnessed a new complete heart block whilst seeing a patient in the ICU and used a shared mental model to work out the cause and plan” (Doctor, Participant 4, Q2)*

*“When I had to transfer a patient with abdominal pain from ED... the pain was so severe that they were having difficulty answering my questions, so I escalated my concern for the patient’s safety. I later went back to review once ED (clinicians) provided analgesia and we were happy the patient had stabilised” (Nurse, Participant 12, Q2)*

### Self-assessment

An overwhelming majority of doctors (35/37 participants) cited that successful workplace learning translation would be self-measured-identified only by internalised markers like improved emotional regulation, recall and use of self-reference guides or different thinking patterns.

*“Being calmer thinking of wider differentials. Not narrow view.” (Doctor, Participant 4, Q1)*

Only nursing participants proffered that they may seek external validation of clinical practice change from senior peers.

*“I will know from better patient outcomes and feedback from supervisors” (Nurse, Participant 5, Q1)*

Curiously, after returning to the workplace post-SBCEE, only nurse participants reported external feedback as a marker of whether their learnings had been harnessed in their clinical practice.

*“On the ward a patient had a clinical review for reduced GCS (Glasgow Coma Scale) so the HMO (House Medical Officer) and I used the systematic method to determine it was likely from aspiration pneumonia and so we organised investigation and management things like oxygen and fluids to stabilise the patient. We also worked with nursing staff to get their impression of the patient’s condition and communicated our plan so they could help us manage the patient. I noticed that I responded to this emergency more quickly and with a clearer approach than before. I am using the session's teachings well” (Doctor, Participant 39, Q1)*

*“At MET [medical emergency] calls where I am not familiar with the patient history, I am going back to the basics as practised during simulations to ensure the emergency priorities are assessed and optimised as best I can. I asked my ANUM [supervisor] and other nurses who attended the call to give me feedback - and this was good, so I am comfortable that I am doing well [translating these learnings to the workplace]” (Nurse, Participant 16, Q1)*

### Successful confidence

The focus of most (184/206) participants simulation learning translation was “confidence”. Participants consistently prized “confidence” as the greatest change to their clinical practice that could be gained from an SBCEE.

“*I hope I will be able to perform more confidently in scenarios where I may need to stabilise unconscious patient” (Doctor, Participant 11, Q1*)

This idea of “confidence” gained from an SBCEE varied widely between participants, regardless of craft group, seniority in clinical experience and workplace setting. Some championed its use in facilitating better communication, others in teamwork or wielding medical equipment.

*“Confidence in being first responder to MET call and being able to initiate basic airway management” (Doctor, Participant 54, Q1)*

Many learners viewed that this “confidence” would change how their minds and emotions responded during a challenging experience; that it would reduce their cognitive load, improve recall, eliminate anxiety, and the ability for observes to perceive their anxieties.

*“I will know that I have translated the learnings to the workplace because the skills will just come naturally, and I will feel more at ease.” (Nurse, Participant 41, Q2).*

Even after returning to the workplace, many participants still viewed “confidence” as the key indicator of mastery in implementing simulation learnings.

*“Yes [I have had the opportunity to use the learnings in my workplace] and will continue to use it to build confidence in fluid resus, workup of sepsis, ordering early investigations and altered conscious state exam.” (Doctor, Participant 30, Q2).*

## Discussion

Taken together, our findings indicate that in our setting, the universal teachings of simulation are variably translated to the workplace and that these variables differ between learners of different craft groups. Whilst most doctor participants felt ill-equipped to use SBCEE learnings in unmatched environments, nursing simulation learners were more readily able to connect SBCEE teachings to novel bedside environments and contexts. Participants' perceived locus of control in simulation translation differed widely, with doctors feeling empowered in their abilities to accurately self-assess and reflect, whilst their nursing colleagues relied on others to provide insight on their clinical performance. This may be due to the differences in how each craft groups learn traditionally [8, 15]; nurses educated to seek corroborative opinions and certification of skills through repeated supervised demonstration, whereas doctors are trained to practice with supervision at a distance, to trial skills in the workplace and make rapid management decisions independently.

Participants from both craft groups perceived that a successful translational simulation resulted in the cultivation of a confidence that afforded enhanced clinical acumen, efficiency and cognitive ease. Interestingly, there were found a great number of differences between craft groups, than commonalities in the transfer of learnings to the bedside (or lack thereof). Overall, our study highlighted the need for SBCEE educators to help learners codevelop workplace translation strategies, facilitate deeper self-reflection and guide the choice of performance indicators.

Translational simulation can be conceptualised operationally as a framework of three phases: input, process and output (IPO) [5]. Despite this, the investment in each phase is not equal with SBCEE educators often only attending to the first two phases [16]. The “input phase” allows the SBCEE setting to be conceptualised with a specific clinical goal in mind and is rigorously evaluated to ensure it is both a feasible and a smart learning investment [5, 16]. The “process phase” affords close collaboration with key stakeholders regarding the design, delivery, assessment and sustainability of an SBCEE [5]. The third and often neglected “output phase requires simulation facilitators to widely report and distribute the analysis of a SBCEE, to ignite workplace change and create new clinical tools and practices supported by these findings [5]. In our setting, limited investment in the “output phase” meant that participants had no infrastructure or point of reference for how SBCEE learnings are usually scaffolded to result in clinical practice change [16]. The imbalance between phases is reflected in the structure of most SBCEEs. For example, time is afforded to engage in experiential activities, experience-informed dialogues on the safety and performance issues that arose during the delivery of the simulation-based interventions, and the purpose of those activities [3]. However, minimal focus is afforded to exploring the application of these learnings across broader contexts, understanding how these learnings can be amplified in different learner settings and facilitating discussions on the granularity with which changes may occur. Making time within simulation learning conversations to address this and continued investment in the “output phase” is necessary to help establish common strategies and pathways for translation simulation [8].

Most doctor participants described a cognitive dissonance between work-based experiences and the types of activities they were primed to use the learnings for during an SBCEE. This contrasts with many nursing participants who recognised their applicability in different settings. Whilst a cause for this craft group difference could not be triangulated, previous studies have proposed the robust psychosocial dimensions afforded by common nursing learning environments compared to those doctors are commonly offered by doctor training [5]. A key pillar of simulation development is careful design to recreate clinical settings that align closely with those experienced by the learner [17] to enhance engagement and immersion by evoking or replicating aspects of the real world that affect clinical performance [1]. This includes designing both the physical and psychosocial dimensions of the learning environment. In the workplace, doctors are often transient and peripatetic members of the clinical team, often working in unfamiliar environments with new equipment, peers, and in general terms, comparatively less time at the bedside. Whereas nurses are more likely to be familiar with the clinical environment, often working in one space (e.g. ward based), with the same peers and perhaps greater patient contact [15]. Thus, in our context, particularly in the setting of COVID-19 restrictions that changed many of our doctors' modes of service delivery and the context surrounding them, the division of doctors from the bedside, the multidisciplinary team and thus contextual clues that help facilitate meaningful clinical learning, may have reduced their abilities to adapt learnings. If this is true, SBCEE workplace translation can be strengthened by investing time during its delivery to explicitly establish the context for learning, providing learners with stepwise guidance on how learnings may be translated [18], and to facilitate learner reflection on the applicability of learnings to other contexts [19].

An overwhelming majority of participants cited that successful workplace learning translation would be self-measured, identified only by internalised markers like improved emotional regulation, recall and use of self-reference guides or different thinking patterns.

Research suggests however that, unfortunately, clinicians may not be the best reporters of their own abilities [20]. Cognitive biases and heuristics form some of the difficulties in accurate clinician self-assessment. This includes a lack of intellectual humility when learning with peers of different standings leading to learners greatly overestimating their knowledge or competence [21]. Yet, extensive peer consultation may be problematic, inaccurate and degrade learner agency [20]. Thus, SBCEE learners should be guided to use a blended approach including objective tools, self-assessment and peer feedback to assess and track simulation translation outcome measures.

A significant finding of the study was the pervasive learner assumption that SBCEE affords opportunities for ‘confidence building’. Moreover, the accrual of confidence was essential to embed in learning, achieve skill mastery, and allow them to feel safe in high-stress environments to explore new learnings and optimise their clinical performance. Whilst participation in an SBCEE can positively affect learners ‘confidence in their knowledge and skills [22], this confidence, in specific settings, has been demonstrated to lead to improved management of acutely ill patients [23], and in other settings, associated with enhanced learner perceived confidence with higher adverse critical events [24]. Similarly, many learners prized the roles of simulation to reduce levels of maladaptive stress during high stakes clinical events and allow them to work more efficiently. The literature to date suggests that the influence of repeated SBCEE exposure on both a clinicians' psychomotor skills and associated stress levels during critical clinical events is negligible [25, 26]. It is also important to acknowledge that adaptive stress can provide impetus for action and enhance efficiency when responding to critical situations [27–29]. Thus, the dichotomous role of stress in clinical performance should always be highlighted during crisis resource management teachings and may not necessarily be a deterrent to the development and consolidation of new skills gained via an SBCEE (Table 2).

**Table 2:** Key recommendations

Key recommendations	
Educators must co-develop simulation translation plans with learners	
Detailing the shared goals of success of a learning	
Above table discussion on the generalisability of learnings to different environments and contexts	
Smart investment in translation simulation outputs	
Cautious championing of learner confidence building	

## Limitations

The accuracy and volume of recalled events may have differed between participants. The study selected a mix of volunteer participants from different work sites, roles, and levels of clinical seniority to capture differences in education impacts experienced by different types of simulation learners. The study did not investigate the characteristics of those who did not volunteer. We can postulate that several factors may have contributed to non-participation including role busyness limiting time for participation, disconnection from the hospital learning community, perceived failure in learning translation and lack of agency. Fundamentally, a limitation of this study is that the data collected represents the subjective learner's perspective. Although learners are often able to predict how well they will remember and follow through with an intention (such as simulation translation), their metacognitive abilities are not veridical, and learners are often overconfident in their predictions and underconfident in post-dictions (more aware of their memory failures than their memorial successes and thus undervalue prior achievements, such as remembering and using previous learning) [28]. As participants attended one of 16 different SBCEEs, the education artefacts (different themes, activities, and learner mix of each event) may have variably impacted upon learners’ abilities to translate learnings into workplace practices. However, all SBCEEs had multiple learnings and were tailored to learner needs (i.e. learners were only admitted to an event if the teachings were deemed relevant to their clinical practice). The questionnaire type provided for free-form answers to allow participants to reflect Future research should explore, in granularity, the effectiveness of different strategies to support learners of different craft groups translating simulation learnings into practice improvement. This may include nuanced approaches to change priming, the delivery of an SBCEE and follow-up post-learning, to ensure the clinical benefit from simulation learning is shared equally amongst interdisciplinary learners.

## Conclusions

Translational simulation is an emerging and promising strategy for improving health service performance and patient outcomes. However, a diverse range of factors influence healthcare workers’ experiences in translating simulation learnings to their workplace. To equip simulation learners to translate learnings from a SBCEE into their clinical practices we suggest the following areas of focus: co-development of translation plans with learners during the delivery of an SBCEE including the indicators of success, the development of shared goals of success, above table discussions on the generalisability of learnings to different environments and contexts, smart investment in translation simulation outputs, and cautious championing of confidence-building. Further evaluation of translation to practice and effect on patient outcomes is warranted in assessing the effectiveness of simulation-based education.

## Data Availability

The datasets generated and analysed during the current study are available from the corresponding author on reasonable request.

## Abbreviations

SBCEE: Simulation Based Clinical Education
